# The complete mitochondrial genome of *Sclerophytum querciforme* Pratt 1903 (Anthozoa: Octocorallia: Sarcophytidae)

**DOI:** 10.1080/23802359.2026.2732536

**Published:** 2026-09-23

**Authors:** Chun-Yang Shen, Wei Xue, Ying Tan, Hao-Yu Shi, Jia-Yi Luo, Xin-Ran Yu, Hui-Fang Guo, Jia-Jia Kong, Yi-Han Yuan, Yan-Fei Wang

**Affiliations:** ^a^ Department of Biology, Chengde Medical University, Chengde, Hebei Province, China; ^b^ Department of Chemical Engineering, Hebei Petroleum University of Technology, Chengde, China

**Keywords:** Mitogenome, Sclerophytum, Sclerophytum querciforme, phylogenetic analysis

## Abstract

Historically, the taxonomic classification and phylogenetic position of *Sclerophytum querciforme*, which is primarily delineated by morphological characteristics, have long remained controversial and undergone repeated revisions. In this study, we sequenced and systematically analyzed the complete mitochondrial genome of *S. querciforme*. The genome is 18,743 bp in length, containing 14 protein-coding genes, two ribosomal RNA genes, and one transfer RNA gene, with the same gene content and order as other species in the genus. Phylogenetic analysis confirmed that *Sclerophytum* forms a well-supported monophyletic clade, and *S. querciforme* is closely related to *S. flexibile* as sister taxa.

## 1. Introduction

*Sclerophytum querciforme* Pratt 1903 is a common soft coral in Indo-Pacific tropical reefs, distributed in the Maldives, Malay Archipelago, New Caledonia, and South China Sea (Pratt 1903; Verseveldt 1980; Benayahu et al. 2018).

This species has experienced repeated generic revisions. It was originally described as *Sclerophytum querciforme* Pratt 1903, and later transferred to *Sinularia* as *Sinularia querciformis* by Thomson and Dean (1931) based on colony morphology and sclerite characteristics. Verseveldt (1980) placed *S. querciformis* in Group IV of *Sinularia*, characterized by clubs longer than 0.12 mm, non-leptoclados-type, and without a central wart. Within this group, *S. querciformis* can be distinguished by loosely arranged lobes and lobules, spiny club heads, and coenenchymal sclerites up to 6 mm long.

However, *Sinularia* shows high morphological plasticity, and intraspecific variation often leads to taxonomic errors (Alderslade and Shirwaiker 1991; Benayahu et al. 1998). For example, *Sinularia elegans* was described based on minor sclerite differences (Tixier-Durivault 1970), but was later synonymized with *S. querciformis* after reevaluation of the holotype (Verseveldt 1974).

Molecular markers such as *mt-mutS* and *COI* have improved the systematics of *Sinularia* (McFadden et al. 2009; Benayahu et al. 2012). Based on recent phylogenomic evidence, *Sinularia* is now restricted to *Sinularia brassica*, and most species, including *S. querciformis*, are restored to *Sclerophytum*, since the valid name of this species is *Sclerophytum querciforme* (McFadden et al. 2022).

Molecular data for *S. querciforme* are still limited to short gene sequences. Mitochondrial genomes (mitogenomes) provide richer phylogenetic data than single genes (Kress et al. 2005; Zhang et al. 2009; Yuan et al. 2012), a pattern also seen in cnidarians including corals (Figueroa and Baco 2014; Colin et al. 2021; Feng et al. 2023). This study sequenced the mitogenome of *S. querciforme* to clarify its phylogenetic position and support future taxonomic and evolutionary studies of octocorals.

## 2. Materials and methods

### 2.1. Sample collection

Specimens of *S. querciforme* were collected by scuba diving from the coast of West Island, Sanya, Hainan Province, China (18°14′5.93″N, 109°22′46.46″E) by Alireza Asem from Hainan Tropical Ocean University. Species identification was based on morphological characteristics, particularly the detailed morphology and size of sclerites, following the descriptions provided by McFadden et al. (2009) and Verseveldt (1980). The colony morphology and sclerite features were illustrated in Figure 1. The voucher specimen is deposited in the Museum of Zoology, Hainan Tropical Ocean University (specimen voucher number: 0058-SQ; Contact: Alireza Asem, asem.alireza@gmail.com).

**Figure 1.**
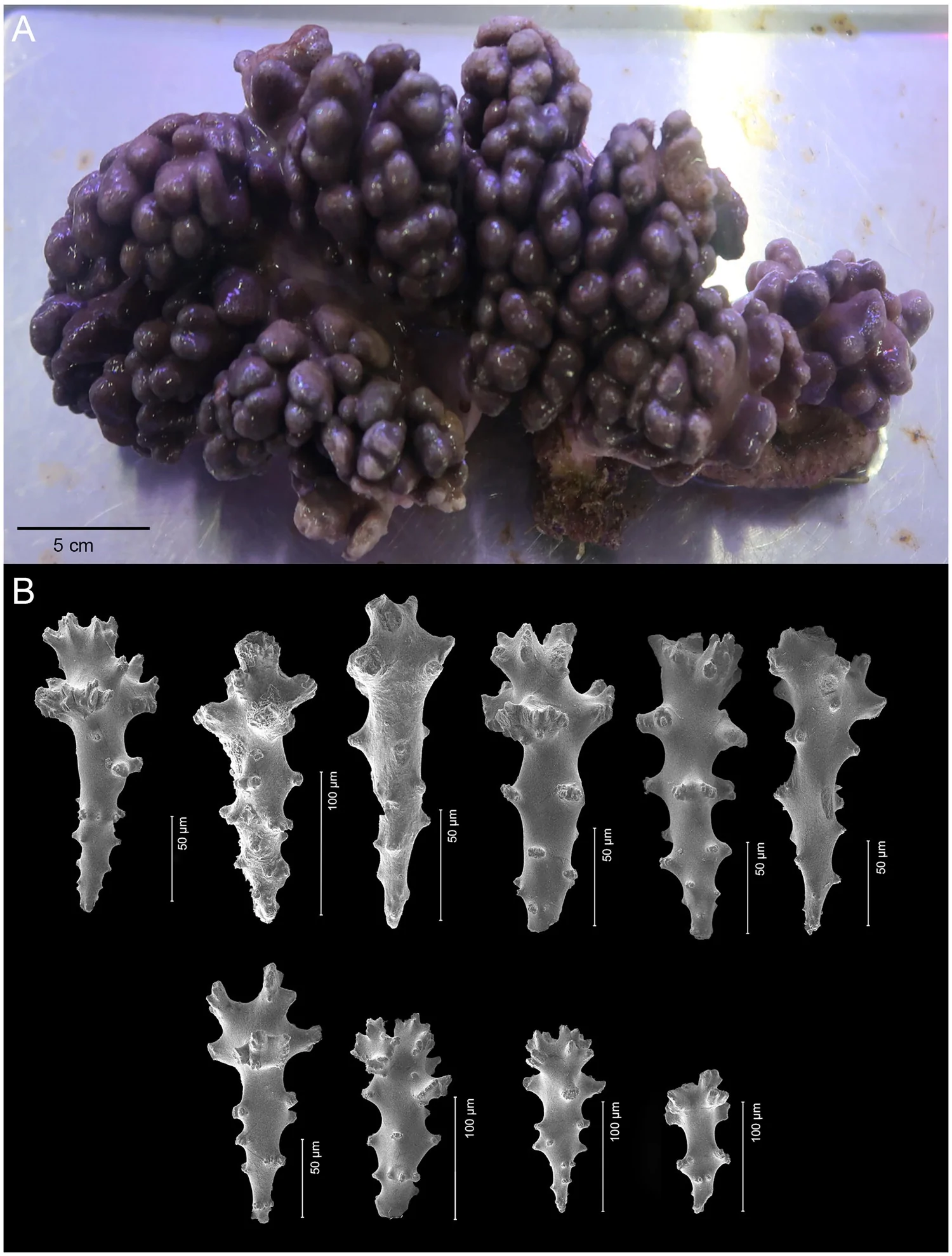
Morphological features of *Sclerophytum querciforme*. (A) Live colony photograph; scale bar = 5 cm. (B) Scanning electron microscopy (SEM) images of sclerites from polyps and the colony surface; scale bars = 50 or 100 μm as indicated.

### 2.2. DNA extraction and sequencing

Genomic DNA of the sample was extracted using the Rapid Animal Genomic DNA Isolation Kit (Sangon Biotech Co., Ltd., Shanghai, China; Cat. No. B518221) following the manufacturer’s standard protocols. A total of 0.2 μg genomic DNA was used for library construction. Genomic DNA was fragmented to 350 bp by sonication, and libraries were prepared using the NEB Next^®^ Ultra^™^ DNA Library Prep Kit (New England Biolabs, Inc., Ipswich, MA, USA; Cat. No. E7370L). Qualified libraries were sequenced on an Illumina HiSeq X platform, generating approximately 10.3 Gb of 2 × 150 bp paired-end clean reads.

### 2.3. Genome assembly and annotation

Raw reads were quality-controlled with FastQC (Andrews, 2010), showing high Phred scores (> Q35) and low error rates (< 0.05%) across all read positions (Figure S1). Reads were filtered with Trimmomatic v0.36 (Bolger et al. 2014) to remove adapters and low-quality reads, yielding clean reads with a 97.92% retention rate. Using the *Sclerophytum ceramense* mitogenome (GenBank: MK292119) as a reference, de novo assembly was performed with GetOrganelle v1.7.7.0 (Jin et al. 2020). Sequencing coverage was quantified after mitogenome assembly, with an average of 771.24×, maximum of 1214×, and minimum of 482× (Figure S2).

Positions of protein-coding genes (PCGs) and ribosomal RNA genes (rRNAs) were determined using the NCBI ORF Finder online server, and manually verified by alignment with homologous genes from related species. Annotation of transfer RNA genes (tRNAs) was performed using tRNAscan-SE Search Server (Chan and Lowe 2019). The mitogenome map was visualized using Proksee (Grant et al. 2023) (Figure 2).

**Figure 2.**
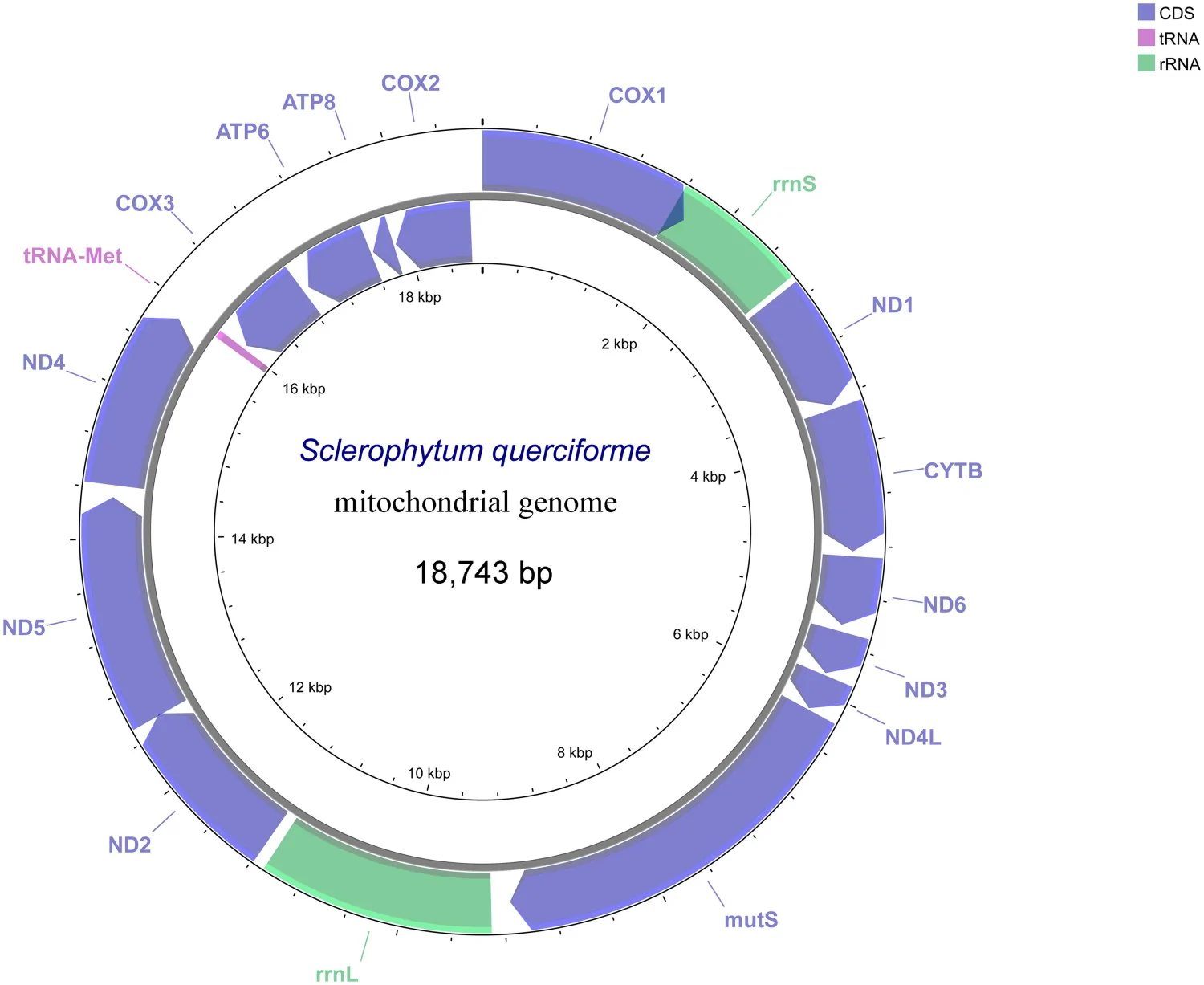
Circular map of the complete mitochondrial genome of *Sclerophytum querciforme*, showing gene locations and types. Genes on the outer track are on the heavy strand, and those on the inner track are on the light strand. Colors indicate gene types (CDS, tRNA, rRNA) as shown in the legend.

### 2.4. Phylogenetic analysis

Phylogenetic analyses included 13 mitogenomes from the genus *Sclerophytum*, with two taxa (*Sarcophyton trocheliophorum and Lateothela grandiflora*) chosen as outgroups in accordance with the octocoral phylogenetic framework (McFadden et al. 2022). Nucleotide sequences of 14 PCGs and two rRNAs were individually aligned in MEGA7 (Kumar et al. 2016) and concatenated into a final 17,755 bp alignment using SequenceMatrix (Vaidya et al. 2011).

Maximum-likelihood phylogeny was constructed using IQ-TREE 2.4.0 (Minh et al. 2020), with all sequences passing the nucleotide composition chi-square test (*p* > 0.05). ModelFinder Plus embedded within IQ-TREE selected GTR+F + I + G4 as the best-fit substitution model according to BIC (Kalyaanamoorthy et al. 2017). Nodal support was quantified using 1000 replicates of UFBoot2 ultrafast bootstrap (Hoang et al. 2018).

## 3. Results

### 3.1. Mitochondrial genome characteristics

The complete mitogenome of *S. querciforme* is 18,743 bp long (GenBank accession: PZ377454). Its nucleotide composition comprises 30.31% A, 16.52% C, 19.30% G, and 33.87% T.

The *S. querciforme* mitogenome encodes 14 PCGs, two rRNAs, and one tRNA. The *trnM* together with *COX3, ATP6, ATP8*, and *COX2* is encoded on the light strand, whereas all other PCGs (*COX1, CYTB, ND1–6, ND4L, mutS*) are distributed on the heavy strand (Figure 2). A total of 15 intergenic spacers are detected, ranging from 4 to 154 bp in length. All PCGs initiate with the ATG start codon. Six PCGs (*ND4L, mutS, ND4, COX2*, *ATP6, ATP8*) terminate with TAA, whereas the remaining eight genes end with TAG.

### 3.2. Phylogenetic tree

The phylogenetic tree (Figure 3) exhibited high bootstrap support values for most branches. The outgroup taxa form a well-supported, distinct clade that is clearly separated from all ingroup species of the genus *Sclerophytum*.

**Figure 3.**
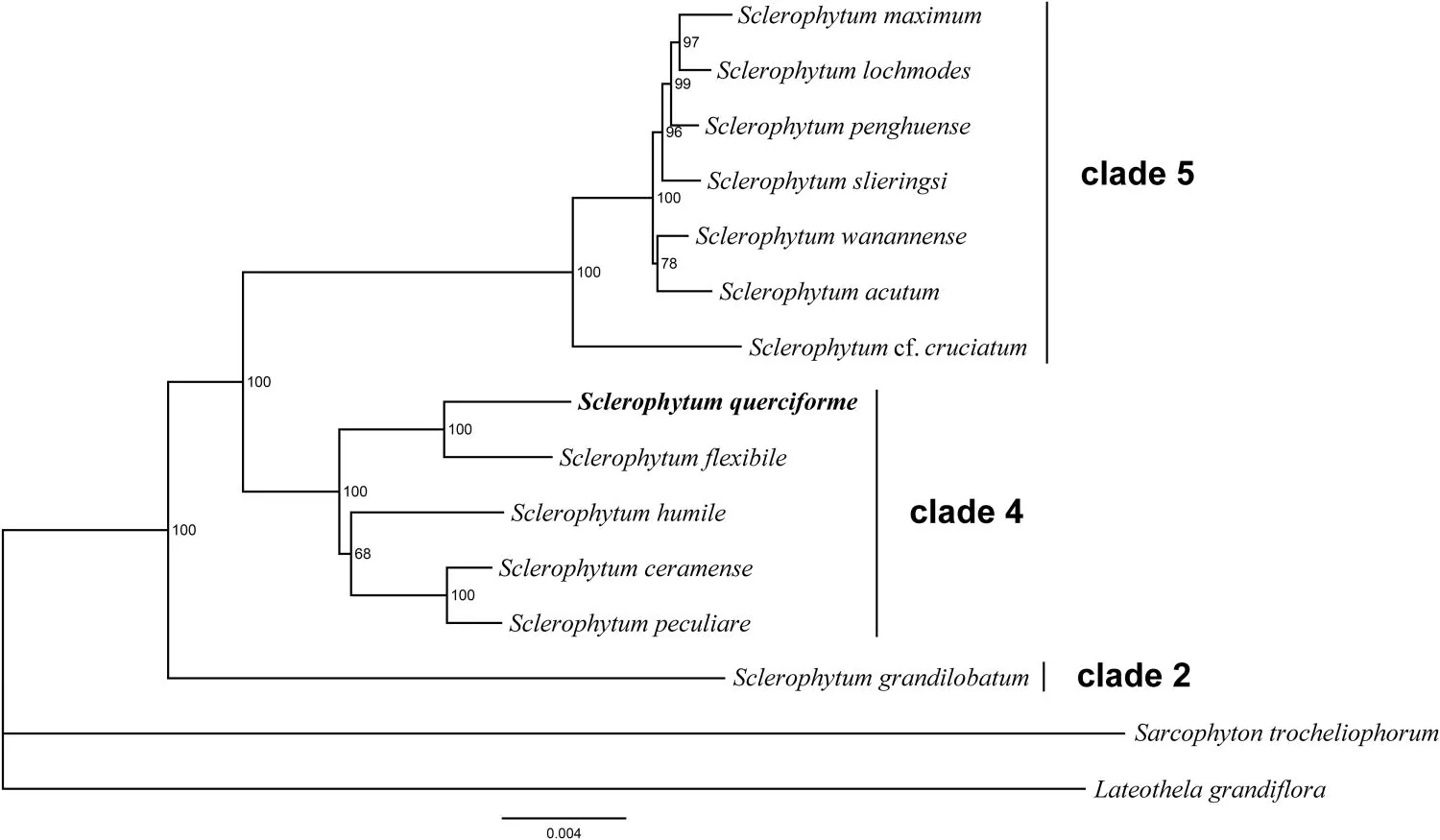
Maximum likelihood (ML) phylogenetic tree of *Sclerophytum* inferred from the concatenated sequences of 14 protein-coding genes and two ribosomal RNA genes. Numbers on branches represent ultrafast bootstrap support values. The scale bar indicates 0.004 nucleotide substitutions per site. The species sequenced in this study, *Sclerophytum querciforme* (PZ377454), is highlighted in bold. Other ingroup species included in the phylogenetic analysis are *Sclerophytum grandilobatum* (PX856750; Shen et al. 2026), *Sclerophytum peculiare* (JX023274; Kayal et al. 2015), *Sclerophytum ceramense* (MK292119; Asem et al. 2019), *Sclerophytum humile* (OK641586; Yang et al. 2022), *Sclerophytum flexibile* (OL616272; Muthye et al. 2022), *Sclerophytum* cf. *cruciatum* (KY462727; Shimpi et al. 2017), *Sclerophytum acutum* (MW987591; Yang et al. 2023), *Sclerophytum wanannense* (OL616277; Muthye et al. 2022), *Sclerophytum slieringsi* (OL616276; Muthye et al. 2022), *Sclerophytum penghuense* (MW256412; Shen et al. 2021), *Sclerophytum lochmodes* (OL616273; Muthye et al. 2022) and *Sclerophytum maximum* (MN485891; Chen et al. 2019). *Sarcophyton trocheliophorum* (MK994517; Shen et al. 2019) and *Lateothela grandiflora* (PX070533; Quattrini et al. 2024) were used as outgroups for rooting the tree.

## 4. Discussion and conclusions

Gene content and gene order comparisons, including the newly sequenced *S. querciforme*, revealed extreme conservation across the genus *Sclerophytum* (Kayal et al. 2015; Shimpi et al. 2017; Asem et al. 2019; Chen et al. 2019; Shen et al. 2021; Muthye et al. 2022; Yang et al. 2022; Quattrini et al. 2024; Shen et al. 2026). This conserved pattern accords with the ancestral gene arrangement of Octocorallia and represents the predominant genomic architecture conserved throughout Octocorallia and its largest order Malacalcyonacea (Brockman and McFadden 2012; Yoshioka et al. 2025).

The phylogenetic tree revealed that the genus *Sclerophytum* formed a strongly supported monophyletic clade, which was divided into three phylogenetic lineages. McFadden et al. (2009) subdivided *Sclerophytum* into five clades (Clades 1–5) using the *muts* gene. Subsequently, Yoshioka et al. (2025) grouped sampled *Sclerophytum* species into two lineages based on available mitochondrial genome data, and assigned them to Clade 4 and Clade 5 following the classification framework of McFadden et al. (2009). Our phylogenetic results are consistent with these previous studies, and the species composition of Clade 4 and Clade 5 perfectly matches the circumscription reported by Yoshioka et al. (2025). Therefore, the three lineages recovered in the present ML tree were designated Clade 2, Clade 4, and Clade 5, respectively.

*Sclerophytum grandilobatum* represented the earliest diverging lineage within the genus, while the remaining species clustered into two well-supported subclades. One subclade comprised *S. querciforme*, *S. flexibile*, *S. humile*, *S. ceramense*, and *S. peculiare*, among which *S. querciforme* was recovered as the sister species of *S. flexibile*.

Mitogenome length within *Sclerophytum* is highly conserved, ranging narrowly from 18,713 bp to 18,752 bp. The mitogenome of *S. querciforme* determined in this study (18,743 bp) falls well within this range. Interestingly, *S. flexibile*—the closest phylogenetic relative of *S. querciforme*—also shares an identical mitogenome length of 18,743 bp, further supporting their close evolutionary affinity.

This study presents the first complete mitogenome of *S. querciforme*. Our results confirm the monophyly of *Sclerophytum*, stabilize its taxonomic status, and reveal highly conserved mitogenomic features within the genus. These findings highlight the value of mitogenome for resolving taxonomic and phylogenetic uncertainties in Malacalcyonacea and provide valuable genomic resources for future studies on soft coral evolution and systematics.

## Supplementary Material

Supplementary Figures S1_S2_The complete mitochondrial genome of Sclerophytum querciforme2.pdf

## Data Availability

The mitogenome sequence data that support the findings of this study are available in GenBank of NCBI under the accession no. PZ377454. The associated BioSample, BioProject, and SRA numbers are SAMN57369155, PRJNA1455540 and SRR38205899, respectively.
