# Supplementary material for: The complete mitochondrial genome of Sclerophytum querciforme Pratt 1903 (Anthozoa: Octocorallia: Sarcophytidae)
Source: Mitochondrial DNA B Resour. 2026 Sep 23;11(10):1228–33. doi: 10.1080/23802359.2026.2732536 (PMC13618110; doi:10.1080/23802359.2026.2732536)
Supplement: Supplementary file 1 [file TMDN_A_2732536_SM4751.pdf]

The complete mitochondrial genome of *Sclerophytum querciforme* Pratt, 1903 (Anthozoa: Octocorallia: Sarcophytidae)

Chun-Yang Shen[a]*, Wei Xue[b], Ying Tan[a], Hao-Yu Shi[a], Jia-Yi Luo[a], Xin-Ran Yu[a], Hui-Fang Guo[a], Jia-Jia Kong[a], Yi-Han Yuan[a], Yan-Fei Wang[a]

[a]Department of Biology, Chengde Medical University, Chengde 067000, Hebei Province, China.
[b]Department of Chemical Engineering, Hebei Petroleum University of Technology, Chengde 067000, China.

CONTACT shenchunyang@cdmc.edu.cn Department of Biology, Chengde Medical University, Chengde 067000, Hebei Province, China.

Supplementary figures (Figure S1 and Figure S2) for the manuscript: The complete mitochondrial genome of *Sclerophytum querciforme* Pratt, 1903 (Anthozoa: Octocorallia: Sarcophytidae)

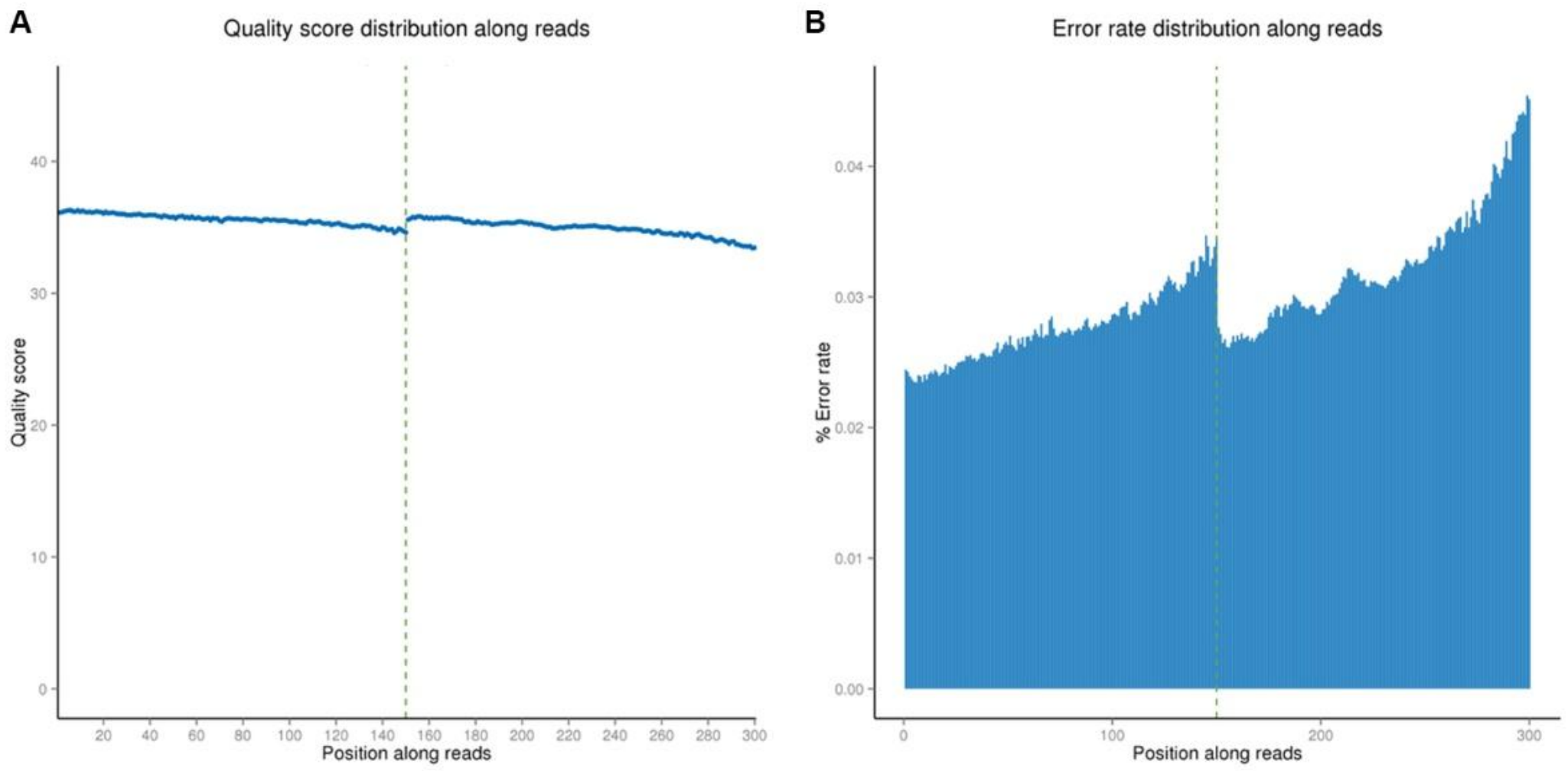

Figure S1. Sequencing quality assessment of raw reads. (A) Quality score distribution along read positions. (B) Error rate distribution along read positions. In both panels, the green dashed line indicates the R1/R2 boundary at approximately 150 bp.

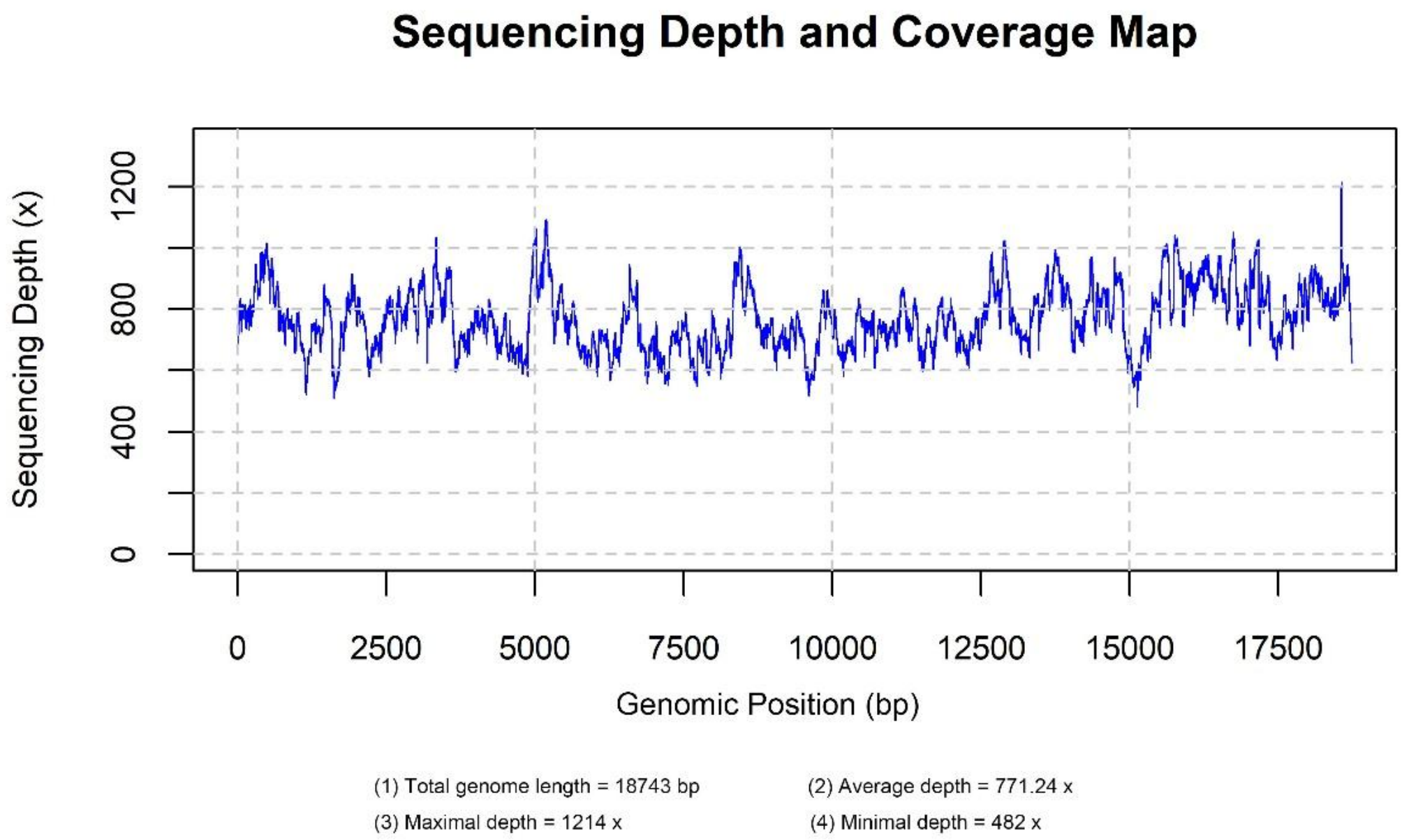

Figure S2. Sequencing depth distribution and coverage metrics across the complete mitochondrial genome assembly of *Sclerophytum querciforme*. The plotted line shows sequencing depth across genomic positions. Indicated values are total genome length = 18,743 bp, average depth = 771.24×, maximal depth = 1214×, and minimal depth = 482×.
